# Analysis of factors associated with the failure of treatment in thoracolumbar burst fractures treated with short-segment posterior spinal fixation

**DOI:** 10.1186/s13018-023-04190-w

**Published:** 2023-09-15

**Authors:** Ehsan Alimohammadi, Seyed Reza Bagheri, Benson Joseph, Hasti Sharifi, Bita Shokri, Lida Khodadadi

**Affiliations:** 1 Department of Neurosurgery, Kermanshah University of Medical Sciences, Imam Reza Hospital, Kermanshah, Iran; 2 Department of General Surgery, University of Tennessee Health Science Center, Memphis, USA; 3 Clinical Research Development Center, Taleghani and Imam Ali Hospitals, Kermanshah University of Medical Sciences, Kermanshah, Iran; 4 Kermanshah University of Medical Sciences, Kermanshah, Iran

**Keywords:** Thoracolumbar burst fractures, Short-segment posterior spinal fixation, Failure of treatment, Load sharing classification, Interpedicular distance

## Abstract

**Background:**

The treatment of thoracolumbar burst fractures continues to pose challenges. Although short-segment posterior spinal fixation (SSPSF) has shown satisfactory clinical outcomes, it is accompanied by a relatively high rate of treatment failure. This study aimed to assess factors associated with treatment failure in thoracolumbar burst fractures treated with SSPSF.

**Methods:**

The clinical data of 241 consecutive patients with a traumatic thoracolumbar burst fracture who underwent SSPSF at our center between Apr 2016 and Apr 2021 were retrospectively reviewed. Patients were divided into two groups (failure of the treatment group and non-failure of the treatment group). We compared potential risk factors for the failure of treatment including age, gender, body mass index, smoking, diabetes, vertebral body compression rate, use of crosslinks, percentage of anterior height compression, presence of index level instrumentation, Cobb angle, interpedicular distance (IPD), canal compromise, Load Sharing Classification (LSC) score, use of posterolateral fusion, and pain intensity between the two groups.

**Results:**

A sum of 137 (56.8%) males and 104 (43.2%) females were enrolled where the mean age and follow-up of the participants were 48.34 ± 10.23 years and 18.67 ± 5.23 months, respectively. Treatment failure was observed in 34 cases (14.1%). The results of the binary logistic regression analysis revealed that the lack of index level instrumentation (OR 2.21; 95% CI 1.78–3.04; *P* = 0.014), LSC score (odds ratio [OR] 2.64; 95% confidence interval [95% CI], 1.34–3.77; *P* = 0.007), and IPD (OR 1.77; 95% CI 1.51–2.67; *P* = 0.023) were independently associated with a higher rate of failure of treatment.

**Conclusions:**

The findings of this study revealed that increased rates of treatment failure in thoracolumbar burst fractures treated with SSPSF were associated with factors such as the absence of index level instrumentation, higher LSC scores, and larger IPD. These findings could be helpful in the proper management of patients with unstable thoracolumbar burst fractures.

## Introduction

Thoracolumbar burst fractures are relatively common spinal injuries [1, 2]. They are characterized by the disturbance of both anterior and middle columns of the vertebral body, resulting in loss of height and widening, constituting close to 20% of all thoracolumbar fractures [3–5]. Although these disruptions can cause varying degrees of spinal deformity, instability and neurologic deficit, the management of these fractures is subject of significant controversy and debate [4, 6, 7]. According to various studies, different strategies have been proposed for the treatment of patients with thoracolumbar burst fractures. These strategies include conservative treatment, anterior surgery, posterior surgery, and a combination of anterior and posterior approaches with the goal of stabilization, maintaining neurologic function and avoiding complications [8].

Short-segment posterior spinal fixation, one level cephalad and one level caudad to the fractured vertebra, has been documented in several studies to yield favorable outcomes in well-selected cases [8, 9]. Reducing the number of instrumented levels offers certain benefits, such as a decreased risk of adjacent segment deterioration and decreased limitations in range of motion [10]. Although SSPSF confers numerous advantages such as less implants, ease of application and a smaller incisions, it can be associated with a relatively high rate of treatment failure where some studies have reported significant rates of implant failure ranging from 9 to 54% [8].

The failure of treatment could be attributed to many factors including the lack of anterior support, implant failure, loss of correction, incomplete decompression of neural elements, inadequate reduction in vertebral body height and biomechanical failure due to the lack of robust instrumentation [10–12]. Nevertheless, SSPSF remains the preferred treatment of choice for the treatment of thoracolumbar burst fractures due to the acceptable clinical and radiological outcomes. The objective of this study was to assess the factors linked to treatment failure in patients with thoracolumbar burst fracture who underwent SSPSF.

## Methods

A total of 241 consecutive patients with a single-level traumatic thoracolumbar burst fracture who underwent short-segment posterior spinal fixation (SSPSF) in our center between Apr 2016 and Apr 2021 were retrospectively evaluated and included in the present study.

We excluded patients with pathologic/osteoporotic fractures. Patients with a history of previous surgery and those with multiple vertebral fractures were excluded too.

The present study was approved by the Scientific Research Board of the Kermanshah University of Medical Sciences. Informed written consent was obtained from all patients before enrollment.

A complete physical examination was performed for all patients on admission to the emergency department. The visual analogue scale (VAS) was used to assess back pain intensity. Non-opioid (paracetamol and non-steroidal anti-inflammatory medicines) and opioid analgesics with or without adjuvant therapies were used for pain management. Analgesia requirements adjusted according to the World Health Organization (WHO) analgesic ladder [13].

We performed preoperative and postoperative anteroposterior and lateral thoracolumbar radiographic studies, thoracolumbar CT scan, T1- and T2-weighted images, and short-tau inversion-recovery (STIR) sequences for all patients. In each subject, the integrity of the posterior ligamentous complex (PLC) was assessed using the STIR sequence [14].

Thoracolumbar Injury Classification and Severity Score (TLICS) was utilized to assess the severity of injury in all patients [15]. Patients with TLICS score > 4 were candidate for surgery.

Indications for SSPSF were patient specific and based on clinical and radiological findings.

We used the traditional SSPSF for all patients. Cord decompression was performed in patients with neurological deficits.

Load sharing classification (LSC) was determined for each patient. This classification assesses three aspects of the fracture, the extent of comminution of the vertebral body, the degree of kyphosis correction achieved post-surgery, and the collapse of the vertebral body in the sagittal plane. Each factor was subdivided into three grades and was scored on a point system from 1 to 3, the sum of the three grades were utilized [16].

**Fig. 1:**
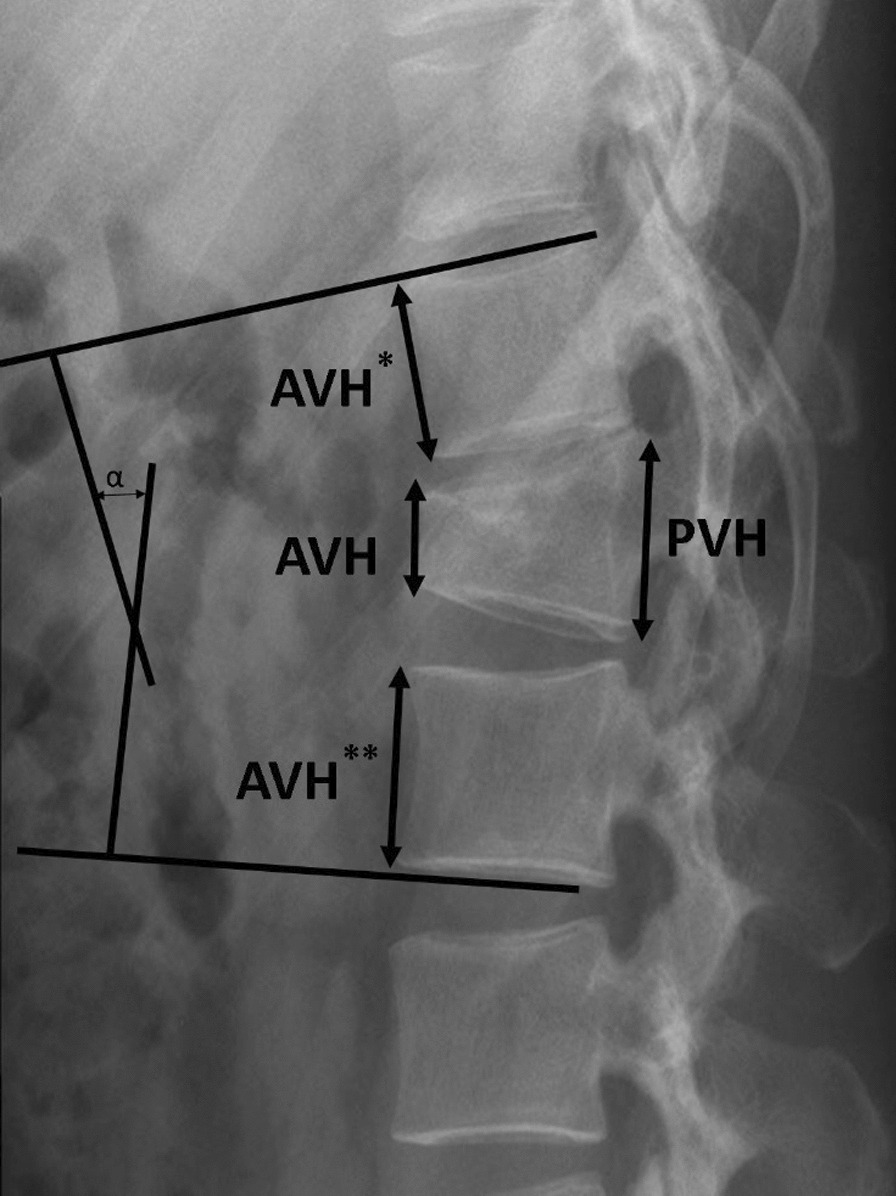
Cobb angle was measured as the angle between the superior endplate of the vertebra above the fracture and the inferior endplate of the vertebra below the fracture. Vertebral body compression rate (VBCR) and percentage of anterior height compression were calculated as follows: VBCR = AVH/PVH × 100%. PAHC = AVH/[(AVH* + AVH**)/2] × 100%. AVH: Anterior vertebral height of the fractured vertebra. AVH*: Anterior vertebral height of a vertebra above the fracture. AVH**: Anterior vertebral height of a vertebra below the fracture. PVH: posterior vertebral height of fractured vertebra

**Fig. 2:**
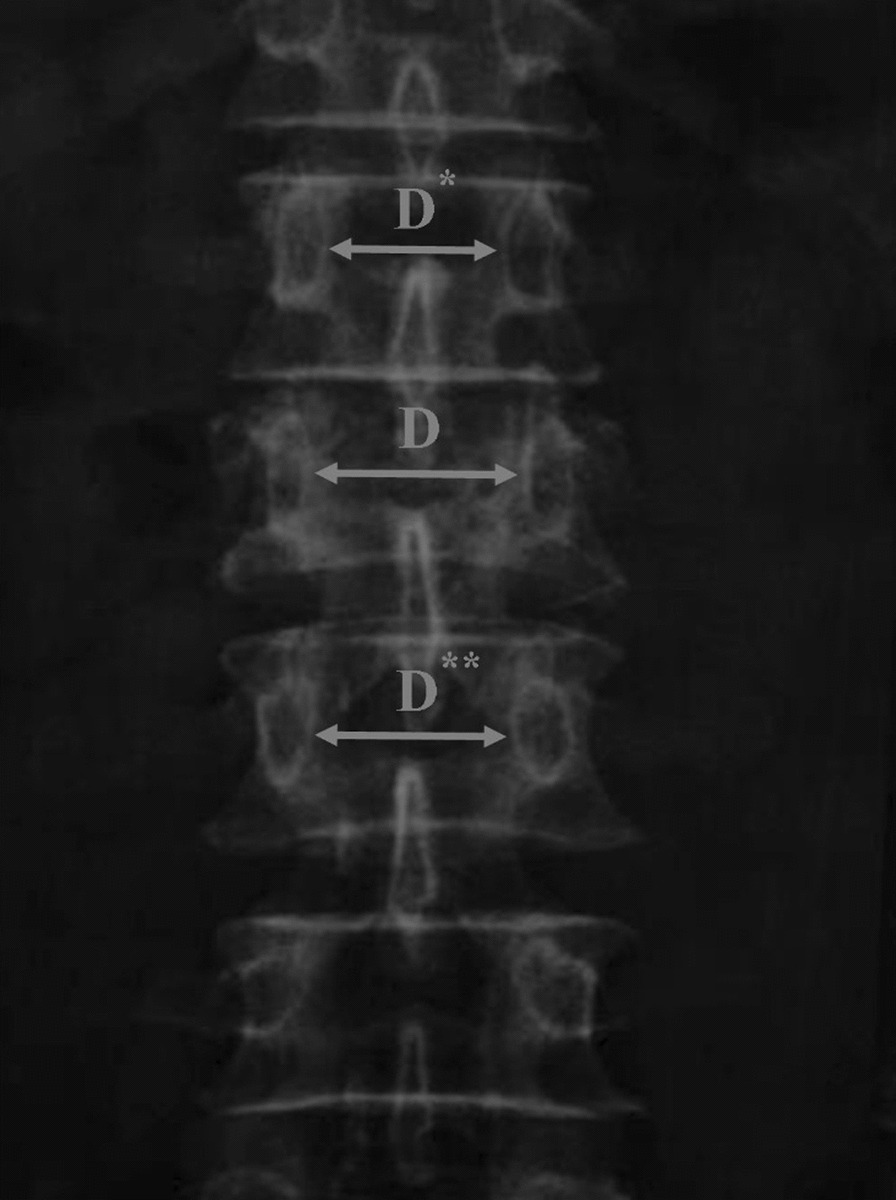
The interpedicular distance (IPD) was calculated by comparing the widening between the pedicles of the fractured vertebrae with the mean of similar values obtained from levels above and below them; IPD = [2D − (D* + D**) / (D* + D**)] × 100%

The following radiological parameters were computed: Cobb angle, canal compromise, interpedicular distance (IPD), vertebral body compression rate (VBCR), and percentage of anterior height compression (PAHC). Cobb angle was calculated as the angle between the two tangents of the upper and lower endplates of vertebra above and below the fracture as shown in Fig. 1 [17]. Canal compromise was measured as the ratio of the spinal canal diameter at the index level to the average of the spinal canal diameter of one vertebrae above and below the fractured vertebra [18]. The interpedicular distance (IPD) was measured by comparing the distance between the pedicles of the index vertebra with the mean distance between the pedicles of adjacent vertebrae above and below the fracture as shown in Fig. 2 [19].

Vertebral body compression rate and percentage of anterior height compression were calculated as follows [20, 21] (Fig. 1):

$$\begin{aligned} {\text{VBCR}} & = {\text{Anterior vertebral height of the fractured vertebra}}/{\text{posterior}} \\ & \quad \quad \quad {\text{vertebral height of the fractured vertebra}} \times {1}00\% \\ {\text{PAHC}} & = {\text{Anterior vertebral height of the fractured vertebra}}/[{\text{Anterior}} \\ & \quad \quad \quad {\text{vertebral height of a vertebra above the fracture}} \\ & \quad \quad + {\text{Anterior vertebral height of a vertebra below the fracture}})/{2}] \times {1}00\% . \\ \end{aligned}$$

The failure of treatment was defined as the presence of instrument failure (i.e., bending of screws, rod fracture, and breakage of screws) and/or progressive kyphosis based on Cobb angle during the follow-up period [18]. Patients were divided into two groups (failure of treatment group and non-failure of treatment group). The potential risk factors for failure of treatment were compared between the two groups.

### Statistical analysis

Data analysis was completed utilizing SPSS 23 software (SPSS Inc. Chicago, Illinois) to analyze the data. We presented data as mean ± standard deviation. The Student’s *t*-test and the Chi-square test were used for comparing continuous and categorical variables between the failure of treatment and non-failure of treatment groups. We conducted a binary logistic regression analysis to assess for independent risk factors associated with failure of treatment. The significance level for the analytical tests was *p* < 0.05.

## Results

A total of 241 subjects treated with SSPSF for thoracolumbar burst fracture were studied (aged 19–65). There were 137 (56.8%) males and 104 (43.2%) females. The mean of age and follow-up time were 48.34 ± 10.23 years and 18.67 ± 5.23 months, respectively. The most common cause of trauma was traffic road accidents (56.4%) and falls (31.1%). As shown in Table 1, the T12 [90 cases (37.3%)] and L1 [77 cases (32.0%)] were the most commonly affected vertebrae. The index level of injury was instrumented in 84 subjects (34.9%) as well, crosslinks were utilized in 69 subjects (28.6%).

**Table 1:** Descriptive characteristics of the sample

Variable		Frequency (%)
Sex	Male	137 (56.8)
	Female	104(43.2)
Failure of treatment	Yes	34(14.1)
	No	207(85.9)
Cause of Injury	Road Traffic crashes	136(56.4)
	Fall	75 (31.1)
	Sport	10 (4.1)
	Assault/violence related	16 (6.6)
	Other	4 (1.7)
Level of vertebra	T10	13 (5.4)
	T11	22 (9.1)
	T12	90(37.3)
	L1	77 (32.0)
	L2	39 (16.2)
Smoking	Yes	47 (19.5)
	No	194 (80.5)
Diabetes	Yes	43(17.8)
	No	198(82.2)
Use of crosslinks	Yes	69 (28.6)
	No	172(71.4)
Index level instrumentation	Yes	84 (34.9)
	No	157(65.1)
Posterolateral fusion	Yes	107(44.4)
	No	134(55.6)

Treatment failure occurred in 34 cases (14.1%). Instrument failure [25 cases (73.52%)] and progressive kyphosis during the follow-up period [9 cases (26.48%)] were the cause of failure of treatment (Table 1). The long segment posterior spinal fusion was performed for all 34 patients in reoperation. No cases needed a combined anterior–posterior approach (Table 2).

**Table 2:** Mean and standard deviation of quantitative variables

Variable	Mean	Standard deviation
Age	48.34	10.23
Follow-up	18.67	5.23
Body mass index	24.23	2.17
VBCR (%)	65.21	5.24
PAHC (%)	70.67	5.11
Cobb(°)	13.13	4.18
Canal compromise (%)	22.73	4.62
LSC	5.73	0.83
IPD(%)	18.72	6.21
VAS	5.64	0.72

*VBCR* Vertebral body compression rate, *PAHC* percentage of anterior height compression, *IPD* Interpedicular distance, *VAS* Visual analogue scale, *LSC* Load sharing classification

### Factors associated with failure of treatment by univariate analysis

As shown in Tables 3 and 4, the lack of index level instrumentation, a higher BMI, a greater Cobb angle, greater IPD on admission, and a higher LSC score was correlated with higher risk of failure of treatment according to the univariate analysis (*p *< 0.05).

**Table 3:** Relationship between qualitative variables and failure of treatment

Variable		Failure of treatment		Statistical analysis
		Yes *N* (%)	No *N* (%)	
Sex	Male	22 (16.1)	115 (83.9)	*P* = 0.843
	Female	12 (11.5)	92(88.5)	
Cause of Injury	Road Traffic	22 (16.2)	114 (83.8)	N/A
	Fall	11(14.7)	64 (85.3)	
	Sport	2(20.0)	8(80.0)	
	Assault	3 (18.7)	13 (81.3)	
	Other	0(0.00)	4 (100.0)	
Level of vertebra	T10	3(23.1)	10(76.9)	N/A
	T11	5 (22.7)	17(77.3)	
	T12	13(14.4)	77(85.6)	
	L1	8 (10.4)	69 (89.6)	
	L2	5(12.8)	34 (87.2)	
Smoking	Yes	7(14.9)	40(85.1)	*P* = 0.159
	No	27 (13.9)	167 (86.1)	
Diabetes	Yes	10(23.3)	33 (76.7)	*P* = 0.214
	No	24 (12.1)	174 (87.)	
Index level instrumentation	Yes	5(6.0)	79 (94.0)	***P *= 0.003**
	No	29 (18.5)	128 (81.5)	
Posterolateral fusion	Yes	19(17.8)	88 (82.2)	*P* = 0.163
	No	15 (11.2)	119 (88.8)	
Use of crosslinks	Yes	11(15.9)	58 (84.1)	*P* = 0.433
	No	23 (13.4)	149 (86.6)	

Bold value indicates* p* < 0.05

**Table 4:** Relationship between need for surgery and failure of treatment

Variable	Failure of treatment		Statistical test
	Yes	No	
Age (year)	49.33 (10.09)	47.01 (9.4)	*P* = 0.327
Follow-Up	18.12 (4.28)	19.43 (5.39)	*P* = 0.301
Body Mass Index	27.15 (2.47)	23.8 (1.2	***P *= 0.012**
VBCR (%)	64.31 (2.31)	67.82 (2.52)	*P* = 0.216
PAHC (%)	67.75 (3.72)	72.36 (4.21)	*P* = 0.419
Cobb(°)	17.02 (3.22)	11.04 (3.17)	***P *= 0.016**
Canal compromise (%)	24.01 (3.27)	21.53 (4.6)	*P* = 0.174
IPD	27.38 (4.51)	18.76 (2.44)	***P *< 0.001**
LSC	7.49 (0.81)	4.93 (0.74)	***P *< 0.001**
VAS	6.02 (0.84)	5.72 (0.77)	*P* = 0.203

Bold values indicate* p* < 0.05

*VBCR* Vertebral body compression rate, *PAHC* Percentage of anterior height compression, *IPD* Interpedicular distance, *VAS* Visual analogue scale, *LSC* Load sharing classification

There was no clear association between the failure of treatment and age, gender, smoking, VBCR, PAHC, canal compromise, VAS, or the use of crosslinks and posterolateral fusion (Tables 3 and 4).

### Factors associated with failure of treatment by multivariate analysis

Based on the binary logistic regression analysis LSC (odds ratio [OR] 2.64; 95% confidence interval [95% CI] 1.34–3.77; *P* = 0.007), the lack of index level instrumentation (OR 2.21; 95% CI 1.78–3.04; *P* = 0.014), and IPD (OR 1.77; 95% CI 1.51–2.67; *P* = 0.023) was strongly associated with the failure of treatment (Table 5).

**Table 5:** Binary logistic regression analysis

Variables	Odds ratio	95% CI	*P* value
Index level instrumentation	2.21	1.78–3.04	***P *= 0.014**
Load sharing classification	2.64	1.34–3.77	***P *= 0.007**
Body mass index	1.27	0.97–1.54	*P* = 0.412
Cobb (°)	1.44	0.89–1.78	*P* = 0.281
IPD	1.77	1.51–2.67	***P *= 0.023**

Bold values indicate* p* < 0.05

## Discussions

In this study, we determined independent factors associated with the failure of short-segment posterior spinal fixation for the treatment of thoracolumbar burst fractures. Through multivariate analysis, we found a statistically significance increase in failure with lack of index level instrumentation, increased LSC, and higher IPD. Short-segment posterior fixation is the most utilized approach for treating thoracolumbar burst fractures. It involves placing screws in the vertebra immediately above and below the fracture level. The primary objective of posterior spinal fixation for treating thoracolumbar burst fracture is to restore spinal stability, regain proper spinal alignment, and to prevent neurological dysfunction [8, 22]. Numerous studies have reported positive outcomes for SSPSF [8, 9, 23–25]. A proper kyphosis angle correction ranging from 6.2° to 21.4° as well significant improvements in anterior vertebral height ranging from 50 to 100% can be achieved after SSPSF [23, 24]. Fracture reduction may be achieved through including postural reduction, pre-contouring of rods, and cantilever correction [8, 25].

In our present study, a lack of index level instrumentation was associated with a higher rate of instrument failure. The use of index level instrumentation, otherwise known as short-same-segment posterior spinal fixation, improves the effectiveness of SSPSF and reduces the occurrence of instrument failure [9, 26, 27]. In a study conducted by Kanna et al., no instances of implant failure were encountered with SSPF with pedicle screw fixation in the fractured vertebrae. It was concluded that even in injuries with LCS > 7, stable reduction can be achieved with SSPF and index level instrumentation [9]. In a cadaveric model, it was shown that the use of index level instrumentation can increase axial and flexion stiffness by 160% and 84%, respectively, preventing excess motion and maintaining spinal stability [26]. In a comparison of nonsegmental and segmental posterior fixation, Mahar et al., found that screws at the level of the fracture offers improved biomechanical stability and less failure. Although it only reported 1 failure, a limitation to the application of this study is that it utilized 6 cadaveric models and a retrospective review of 12 patients [27].

The utilization of index level instrumentation continues to be a topic of exploration in the treatment of thoracolumbar burst fractures. In the evaluation of 72 thoracolumbar burst fractures treated with SSPSF, Guven et al. concluded that pedicle screw placement into the fractured vertebrae reduced the rates of kyphosis correction failure. Index level instrumentation was found to be associated with a reduced risk of correction loss, although there was no association between loss of kyphosis correction and factors such as load sharing classification, Magerl type, or level of injury [28]. It was suggested that this form of fixation was equivalent long segment with fixation two levels above and below the injured vertebrae [29]. However, Farrokhi et al. [30] observed a higher incidence of implant failure and loss of kyphosis of 29% in cases without index level instrumentation, as in comparison with 6% in patient who underwent index level instrumentation.

A literature review conducted by Tanasansomboom et al. [31] reported that intermediate screw fixation into the fractured vertebra reduces the risk of instrument failure and accelerates bone healing. In a biomechanical model that compared long segment, short segment, and short-segment fixation with same level instrumentation, it was concluded that same level screw fixation of thoracolumbar burst fractures with LSC < 7 resulted in stable fixation, better spinal range of motion, lower correction loss and lower failure rates [32]. Although controversial, many studies have demonstrated the advantage of index level instrumentation and improved clinical outcomes.

In our study, patients with higher LSC scores were associated with a higher rate of treatment failure (OR 2.64; 95% CI 1.34–3.77; *P* = 0.007). The Load Sharing Classification system utilizes the degree of vertebral comminution as a prognostic factor for the failure of instrumentation in patients who undergo SSPSF. In the original study by McCormack et al. [16], they evaluated 28 thoracolumbar fractures treated with SSPSF where all failures had a LSC of > 6 points. Consistently, in a study conducted by Altay et al. [23], fracture location, fracture type and LSC score were all associated with higher correction loss when treated with SSPSF. However, in a retrospective review, Scholl et al. [33] examined a group of 22 patients treated with SSPSF for thoracolumbar fractures and determined that LSC was not a reliable indicator of implant failure.

There is an ongoing debate about the utility of the LCS. In a comprehensive systematic review of 21 studies that there was no significant association between LSC and instrumentation failure [34]. The management of thoracolumbar burst fractures has significantly changed since the introduction of the LSC in 1994, the use of intermediate screws, cement augmentation or titanium result in more robust constructs have changes operative procedures and may alter the utility of the original classification system [34, 35]. A prospective study conducted by Aligizakis et al. [36], cited the lack of ligament damage grading as one reason why LCS is useful in preoperative analysis in determining candidates for SSPSF but is best used as an adjunctive and not individual prognosticator for treatment failure. In a study exploring the efficacy of monosegmental transpedicular fixation, it suggested that LSC ≥ 8 is predictive of failure, as opposed to the originally proposed LSC ≥ 7 [37]. Although LSC is utilized to classify thoracolumbar burst fractures, its utility has changed and continues to be a topic of debate.

Within this study, the increased IPD was associated with higher rate of treatment failure (OR 1.77; 95% CI 1.51–2.67; *P* = 0.023). Previous studies have demonstrated that IPD is associated with spinal canal narrowing by fractured vertebrae, neurologic deficits, and laminar fractures [38, 39]. Caffaro et al. [38], suggested a correlation of IPD and neurologic deficits to be as great as 65%. Increased interpedicular distance is suggestive of the degree of vertebral body involvement. Dong et al. [40], utilized machine learning models to predict adverse events after single segment fixation and concluded that IPD was on of the most important risk factors in predicting treatment failure. Multiple studies describe the process as retrograde bone fragments lead to canal compression that cause more severe neurologic dysfunction [38–40].

In a systematic review and meta-analysis including 601 patients, increased IPD on admission was significantly predictive of failure of treatment. Although this study explored conservative management, it is proposed that canal remodeling offers improvement in canal area [41]. Tanriverdi et al. [42] studied 106 patients with T10-L3 thoracolumbar burst fractures and found higher level of neurologic deficits in patients with higher IPD and canal compromise. It was suggested that the relationship is stronger at levels T12 and L1 [42]. Although there is strong consensus of the influence of IPD on fragment displacement and instrument failure, exploration is needed to explore the relationship between IPD and the failure of SSPSF in the treatment of thoracolumbar burst fractures.

## Limitations

There are several limitations to this study. This was a retrospective single-center study with a relatively small number of cases. The lack of a long-term follow-up is another important limitation of the present study. We recommend multicenter prospective studies with a larger sample size to further evaluate factors associated with the failure of treatment in patients with thoracolumbar fractures who underwent SSPSF.

## Conclusion

This study indicates that index level instrumentation decreases the rate of treatment failure in SSPSF for single-level thoracolumbar burst fractures. The results of the present study also provided strong support that a greater LSC score and a greater IPD may be associated with higher rates of failure of treatment in thoracolumbar burst fracture treated with SSPSF. Short-segment posterior spinal fixation one level above and below the injury remains to be controversial in the treatment of thoracolumbar burst fractures. Although these findings may be helpful in the proper management of patients with unstable thoracolumbar burst fractures, more clinical studies are needed to elucidate their relationship to treatment failure.

## Data Availability

The datasets generated and/or analyzed during the current study are not publicly available due them containing information that could compromise research participant privacy/consent but are available from the corresponding author on reasonable request.
